# Exploring the link between multiscale entropy and fractal scaling behavior in near-surface wind

**DOI:** 10.1371/journal.pone.0173994

**Published:** 2017-03-23

**Authors:** Miguel Nogueira

**Affiliations:** Instituto Dom Luiz, Faculdade de Ciências da Universidade de Lisboa, Campo Grande, Lisbon, Portugal; CNRS, FRANCE

## Abstract

The equivalency between the power law behavior of Multiscale Entropy (*MSE*) and of power spectra opens a promising path for interpretation of complex time-series, which is explored here for the first time for atmospheric fields. Additionally, the present manuscript represents a new independent empirical validation of such relationship, the first one for the atmosphere. The *MSE*-fractal relationship is verified for synthetic fractal time-series covering the full range of exponents typically observed in the atmosphere. It is also verified for near-surface wind observations from anemometers and CFSR re-analysis product. The results show a ubiquitous *β* ≈ 5/3 behavior inside the inertial range. A scaling break emerges at scales around a few seconds, with a tendency towards 1/f noise. The presence, extension and fractal exponent of this intermediate range are dependent on the particular surface forcing and atmospheric conditions. *MSE* shows an identical picture which is consistent with the turbulent energy cascade model: viscous dissipation at the small-scale end of the inertial range works as an information sink, while at the larger (energy-containing) scales the multiple forcings in the boundary layer act as widespread information sources. Another scaling transition occurs at scales around 1–10 days, with an abrupt flattening of the spectrum. *MSE* shows that this transition corresponds to a maximum of the new information introduced, occurring at the time-scales of the synoptic features that dominate weather patterns. At larger scales, a scaling regime with flatter slopes emerges extending to scales larger than 1 year. *MSE* analysis shows that the amount of new information created decreases with increasing scale in this low-frequency regime. Additionally, in this region the energy injection is concentrated in two large energy peaks: daily and yearly time-scales. The results demonstrate that the superposition of these periodic signals does not destroy the underlying scaling behavior, with both periodic and fractal terms playing an important role in the observed wind time-series.

## Introduction

Intensive research efforts undertaken over the recent decades have provided with robust evidence for the existence statistical scale-invariance (scaling) properties in the complex structure of atmospheric winds (see, e.g., [1, 2, 3] for reviews). Typically, near-surface wind time-series display power-law scaling behavior with a ubiquitous high-frequency inertial range, with a 5/3 scaling exponent in close agreement with Kolmogorov [4] predictions. This inertial range starts at the viscous dissipation scale (on the order of millimeters for the atmosphere), but there is no agreement on its low-frequency boundary. On the one hand, several previous works have found an inertial range with nearly constant spectral exponent *β* ≈ 5/3 up to a few days (e.g. [5–7]). On the other hand, several other authors reported the inertial range breaking at much shorter time-scales, ranging between a few seconds to minutes (e.g. [8–13]). Most of these latter works found a transition to a *β* ≈ 1 − 1.3 regime after the break. At even larger scales, a rather generalized consensus exists on scaling transition. It occurs at scales between about 1 and 10 days, with an abrupt flattening of the power spectra and the emergence of scaling regime with low spectral exponents. This low-frequency regime is commonly designated by “spectral plateau” due to the abrupt decrease of the spectral slope, with *β* values often close to zero. However, as pointed out in [7] this is somewhat of a misnomer since it is clear that the regime has a nonzero logarithmic slope. The alternative denomination of “low-frequency weather” suggested in [7] for this region of the temporal spectrum is adopted here. The extension of this low-frequency weather can vary between different works, ranging from a few to hundreds of years depending on the particular variable and dataset analyzed (see, e.g., [2, 14] for reviews). For example, its spectral exponent and scale range have been found to be dependent on latitude and on the land-sea transition (e.g. [14, 15]). Nonetheless, the existence of pronounced flattening at scales larger than a few days seems to be a ubiquitous property of atmospheric winds.

Consequently, the shape of a typical temporal wind spectrum in the atmospheric boundary layer (ABL) is still matter of debate and the problem of whether the scaling exponent for a given atmospheric field and the respective scaling range are universal (constant) properties or instead dynamically dependent on the “local” environmental conditions remains largely open. In fact, there is a growing number of studies proposing linkages between the different scaling exponents and the particular atmospheric conditions (e.g. atmospheric stability, mean wind speed) and surface forcings (orography and surface fluxes) [16–19].

Another commonly used framework used to investigate complex signals with variability over a broad range of scales, is the Multiscale Entropy (MSE), first introduced by [20]. MSE is based on the concept of information entropy, *H*(*X*), introduced in Shannon's seminal 1948 paper [21]. It measures to what extent does the next character in a message, *X*, provides with new information. In the discrete case *H*(*X*) is given by:

$$H(X)=-\sum_{x_{i}\epsilon \theta }\;p(x_{i})\;\log\;p(x_{i})$$ (1)

where *θ* is the set of all values that *X* can assume and *p*(*x*_*i*_) = Pr[*X* = *x*_*i*_] is the respective probability distribution. Note that by convention *p*(*x*_*i*_) log *p*(*x*_*i*_) = 0 if *p*(*x*_*i*_) = 0. If the next value in a time-series is always known with certainty, i.e. *p*(*X* = *x*_*i*_) = 1 (e.g. periodic signal), then *H*(*X*) = 0. Conversely, in a random system the next observation is unexpected and we must keep observing the system to see how it is evolving. This can be illustrated by the flip of a fair coin (*p*(*heads*) = *p*(*tails*) = 1/2) where a higher information entropy value *H*(*X*) = −2(0.5 log 0.5) = 0.69 is obtained. Extending for *n* equiprobable outcomes it is easy to show that *H*(*X*) = log(*n*), implying that entropy (uncertainty) increases with the number of equiprobable outcomes.

A great advance provided by information entropy lies in the discovery of a unique unambiguous criterion for the “amount of uncertainty” in a time-series, which agrees with the intuitive notion that a broad probability distribution represents more uncertainty than does a sharply peaked one, and satisfies all other conditions which make it reasonable [21, 22]. Inspired by Shannon's information entropy, Sinai generalized this concept of entropy for measure-preserving dynamical systems [23]. He defined the so-called Kolmogorov-Sinai entropy (KS) which measures the mean rate of creation of information with each new state of a dynamical system, and hence also measures to what extent we are able to predict the next step in the sequence. Essentially, the state of a system at a certain time instant *X*(*t*_*i*_) is partially determined by its history *X*(*t*_1_),*X*(*t*_2_),…*X*(*t*_*i*−1_). Higher KS entropy implies a higher amount of new information being introduced at the next time-scale and, hence, higher unpredictability of the dynamical system.

Direct estimates of the KS entropy were shown to be of limited use to estimate the entropy of finite length and noisy “real world” time-series (e.g., [24, 25]). In order to overcome this problem, [24] introduced the concept of approximate entropy, *A*_*E*_, a family of measures of regularity closely related to KS that can be applied to these short and noisy time-series. Subsequently, [25] defined the sample entropy, *S*_*E*_, a refinement of *A*_*E*_ which they showed to agree with theory more closely, to be less dependent on time-series length, to show relative consistency over a broader range of parameter values and to be computationally more efficient.

A limitation of measures such as *A*_*E*_ and *S*_*E*_ is that they only consider a single scale, the shortest scale of the time-series. Consequently they are highly dependent on the measuring resolution of the data-set and they are inadequate to fully characterize the multiscale structure of turbulent atmospheric processes [20, 26]. Additionally, there is no straightforward general correspondence between these single-scale entropy measures and complexity (see e.g. [20, 27]). Neither completely predictable signals (e.g. regular and periodic), which have minimum entropy, nor completely unpredictable random signals (e.g. uncorrelated white noise), which have maximum entropy (dependent on the signal length) are structurally complex, since they admit a very simple and compact description [27, 28]. A meaningful complexity measure, therefore, should vanish for these two extreme states.

In order to overcome these limitations, *MSE* was introduced by successively computing the sample entropy after coarse-graining the original time-series at different temporal scales using a moving average window [20, 27]. Using *MSE* analysis, they pointed out that one can clearly see that although at scale one (time-series sample period) uncorrelated white noise has a higher value of entropy than a correlated 1/f (scaling) noise, when increasing scales are considered the entropy of the white noise decreases monotonically towards zero and rapidly falls below the entropy value of the 1/f noise, which remains constant with scale. They argue that correlated random signals are more complex than uncorrelated ones, in the sense that the former contain complex structures across multiple time scales whereas the latter does not.

Since its introduction, *MSE* has been successfully applied in different research fields, including physiological, financial, seismic and hydrological time-series (see e.g. [29] for a recent review). These previous investigations have demonstrated the value of *MSE* to study complex signals with variability over wide ranges of scales. But the potential of *MSE* for the particular case of atmospheric wind time-series has been scarcely explored. [30] applied MSE analysis to near-surface wind data from a 3-D ultrasonic anemometer. They found that low, moderate and intense wind regimes displayed different variation of the entropy with scale on the sub-second scales. [31] applied *MSE* analysis to horizontal wind time-series in the ABL, at temporal scales between 0.25 s and 5 s. Their results showed a similar picture for both zonal and meridional components: entropy increasing with scale, with a steep increase rate at the small scales and then becoming flatter at the large scales. Similar results were obtained in [13], where the normalized Shannon's entropy was estimated directly from turbulent vertical wind time-series at different temporal scales and relevant differences between vertical velocity time-series with nearly stationary or non-stationary increments were reported. *MSE* analysis has also been applied to other atmospheric and hydrological variables including temperature, rainfall and surface runoff over different ranges of scales ([32–35]).

Several previous works have related the *MSE* variation of a 1/f noise with its scaling exponent (e.g. [20, 26, 27]). More recently, [36] empirically found that this can be generalized for fractional Gaussian noises with spectral scaling exponents *β* > 0. Their empirical analysis showed that the slope of log-log plots of *MSE* against temporal scale matched the fractal scaling exponent. They also confirmed this empirical result for electroencephalogram time-series. [37] derived analytically a bi-scaling law that provides a possible explanation for this relationship between *MSE* variation with scale and fractal scaling behavior. They also confirmed this relationship empirically using heart rate time-series.

This type of exact correspondences between seemingly different concepts play important role in all fields of physics. In particular, this equivalence between the power-law exponents of *MSE* against time-scale and fractal scaling exponents opens a new path for interpretation of their scaling behavior based on information theory. However, this path has not been explored for atmospheric time-series and their turbulent cascade properties. In the present manuscript, the connection between the power-law behavior of information entropy against scale and scaling exponents is explored for the first time in the atmosphere, using near-surface wind-time series. The goal is to gain new insights on their complex temporal structure, particularly on the occurrence of scaling breaks, while also providing further empirical proof of the MSE-fractal relationship over a wide range of temporal scales (from a around 50 ms to decadal).

The present manuscript is organized as follows: the data-sets, *MSE* and scaling analysis methodologies are presented in Section 2. The results of the *MSE* and scaling analysis on near-surface wind observations but also on fractal synthetic time-series are presented in Section 3. Finally, the main results are summarized in Section 4.

## Data and methodology

### Scaling analysis

The scaling behavior of atmospheric time-series is commonly investigated using the first order structure function, given by:

$$\langle \left|\Delta f(\Delta t)\right|\rangle =\langle \left|f(t+\Delta t)-f(t)\right|\rangle \propto {\Delta t}^{H}$$ (2)

where the angled brackets denote statistical averaging, Δ*t* is the time lag and *H* is the non-conservation scaling exponent, representing a measure of the degree of smoothness of the process. In the non-conservative case (*H* ≠ 0), the average fluctuations exhibit a scale dependence. The Fourier spectral analysis is an alternative way to investigate the presence of scale invariant behavior in atmospheric fields over a wide range of scales, manifested as log-log linearity of the power spectrum:

$$E(k)∼k^{-\beta }$$ (3)

where *k* is the frequency and *β* is the spectral scaling exponent, which can be obtained by linear square regression on the log-log spectra. Eq (3) represents a simple and commonly used framework that is familiar to atmospheric scientists and is quite sensitive not only to the presence of scaling behavior but also to scaling breaks and other types of deformations of the power law behavior. Neglecting the intermittency corrections, *β* is related to *H* by:

β=1+2H (4)

Notice that Eq 4 represents a first-order approximation, since multifractal behavior is commonly reported for wind time-series which adds a correction term: *β* = 1 + 2*H* − *K*(2) (e.g. [38]). However, here the intermittency corrections are considered second order effects and neglected for the sake of simplicity. It can be seen from Eq (4) that Kolmogorov scaling with *H* = 1/3 corresponds to *β* = 5/3.

### Multiscale entropy analysis

In this section we provide a brief description of the *MSE* algorithm, following [20, 27] where a more comprehensive description can be found. The algorithm can be summarized in two main steps. In the first step, a coarse-graining procedure is applied to a discrete time-series *f*_*i*_ (*i* = 1,…,*N*) in order to construct a new set of time-series representing the system dynamics on different time scales. Each element of the coarse-grained time-series, *f*_*j*_^(*τ*)^, is calculated as the average of *f*_*i*_ over consecutive non-overlapping windows of length *τ*, trimming any residual data points at the end of the time-series:

$$f_{j}^{\left(\tau \right)}=\frac{1}{\tau }\sum_{i=(j-1)\tau +1}^{j\tau }f_{i}$$ (5)

where index *j* varies between 1 and the length of each coarse-grained time-series, *N*/*τ*. At scale *τ* = 1 the coarse-grained time-series is simply the original series (*f*_*i*_ = *f*_*j*_^(1)^).

In the second step, the sample entropy is computed for each coarse-grained time-series *f*^*τ*^. *S*_*E*_ is defined as the negative logarithm of the conditional probability that two similar sequences of *m* consecutive data points remain similar when their length is increased by one point (*m* + 1) [25]:

$$S_{E}(m,r_{T},N)=-\log\frac{A^{m}(r_{T})}{B^{m}(r_{T})}$$ (6)

where *A*^*m*^(*r*_*T*_) is the probability that two sequences will match for *m* + 1 points (with a tolerance of *r*_*T*_) and *B*^*m*^(*r*_*T*_) is the probability that two sequences will match for *m* points (also with a tolerance of *r*_*T*_), where self-matches are excluded. In other words, *S*_*E*_ is a statistical property that quantifies the degree of regularity of a time-series by evaluating the appearance of repetitive patterns [25]. A high degree of regularity (more repetitive patterns) leads to lower values of *S*_*E*_. Conversely, large values of *S*_*E*_ are obtained when the underlying dynamical process produces large irregularities as time advances. In this sense, the sample entropy is also a measure of unpredictability of the signal. Finally, *MSE*(*τ*) is computed by estimating *S*_*E*_ for different coarse-graining scales of the same time-series.

In the *MSE* algorithm two vectors are considered similar if the distance between them is lower than a tolerance parameter, *r*_*T*_, usually chosen as a percentage of the standard deviation of the original time-series, *r*_*T*_ = *r* × *σ*(*f*_*i*_). The distance between two vectors is computed as the maximum absolute difference of their corresponding scalar components. Here *r* values of 0.2 are considered, a common practice based on previous *MSE* based investigations.

The previous works of [36] and [37] have suggested that *MSE* dependence on time-scale is tightly related to the fractal scaling behavior. Here we empirically test the hypothesis that *MSE* displays a power law dependence on scale with the same exponent as the first order structure function of the time-series:

$$MSE(\tau )\propto \tau^{H}$$ (7)

Notice that [37] found that *MSE* is also tightly related to the length of the considered phase space bins (i.e., for a single variable 1-dimensional time-series, the minimum detectable signal). Here we consider the minimum detectable signal to be constant for a given data-set and hence this dependency is neglected.

### Datasets

**Fig 1:**
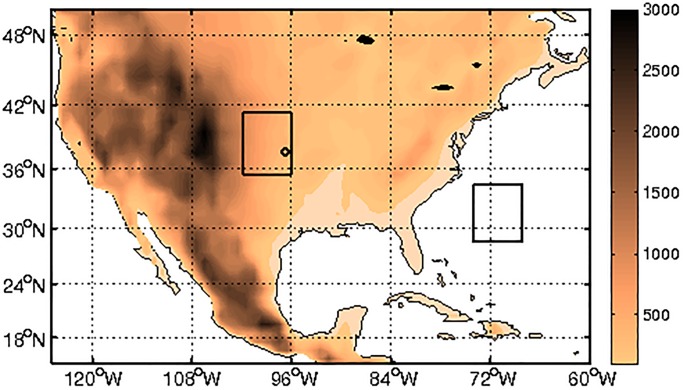
Map of the considered domain. The squares identify the two CFSR sub-domains while the circle identifies the location of the 6 CASES-99 anemometers. The color scale represents the topographical height in meters.

High-frequency near-surface wind data was obtained from the Cooperative Surface-Atmosphere Exchange Study (CASES-99) Integrated Surface Flux System. This field campaign was undertaken during October 1999 over southeast Kansas in the United States (Fig 1). Further details on the CASES-99 project and data-sets can be found in [39]. The 3-dimensional wind components were measured at six different stations with 3-D sonic anemometers mounted 5 m above the ground. The stations were located very close to each other (see Table 1). The data was previously re-sampled to 20Hz time grid and the wind vectors have been rotated to geographical coordinates and corrected for the tilt of the anemometer relative to a plane of mean flow. The data was collected between October/6/1999 and October/31/1999.

**Table 1:** Location of the 6 CASES-99 anemometers.

#	Lat. [°]	Long. [°]
1	37.65893	-96.73507
2	37.64750	-96.73647
3	37.64895	-96.73718
4	37.64983	-96.73870
5	37.64595	-96.73644
6	37.64968	-96.73332

For the present work, the CASES-99 data-set was split into several time-series realizations, each with 2^17^ data points, corresponding to approximately 1 hour and 49 minutes. This particular choice of power of two length of the time-series is convenient for Fourier analysis. Additionally, it is important to notice that in order to obtain reasonable sample entropy values, the coarse-grained time-series length should have at least 10^*m*^ = 100 points. Given the present choice of *m* = 2 for the *MSE* analysis, following [20, 27]), the chosen length of 2^17^ data points allows for the coarse-graining operator to extend from 0.05 s up to 50 s. The decomposition into 2^17^ points time-series also allows to obtain a large “valid” data-set composed by 1205 time-series realizations, created by retaining only those realizations without any missing data points for both the zonal and meridional wind components.

Near-surface zonal and meridional wind time-series representing longer time-scales were obtained from the National Centers for Environmental Prediction (NCEP) Climate Forecast System Reanalysis (CFSR), which provides hourly data gridded at 0.5° × 0.5° resolution. For details on this dataset see [40]. Two sub-domains of 20 × 20 grid points were chosen for the present study: (D1) is located over land, encompassing the region where the CASES-99 stations were located, and (D2) is located offshore, over the Atlantic Ocean close to the Southeastern U.S (Fig 1).

**Fig 2:**
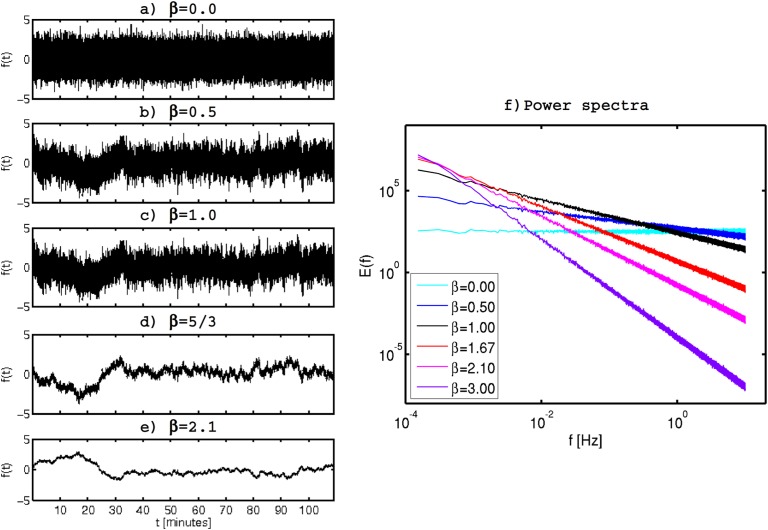
Samples of synthetic fractal time-series all with the same generator but with varying *β* values: a) 0.0, b) 0.5, c) 1.0, d) 5/3 and e) 2.1. f) Log-log ensemble Fourier power spectra averaged over the 60 realizations for each of the considered *β* values.

Finally, synthetic fractal (SF) time-series were built using a fractal generator. The algorithms starts by creating a normally distributed random noise, then transform it into the Fourier domain and impose the desired *k*^−*β*^ scaling on the power spectrum. The inverse Fourier transform is then applied and the result is synthetic fractal (SF) time-series with the desired scaling properties. This process is repeated to generate 60 SF realizations with different generators (i.e. different “random” normally distributed noises) for each of the considered *β* values (0, 0.5, 1, 1.1, 1.3, 5/3, 2.1 and 3). All of the realizations have mean equal to zero, a sample frequency of 20 Hz and a length of 2^17^ data points. Five samples of SF time-series with different scaling exponents are shown in Fig 2A–2E, illustrating the effect of the variable fractal exponent on signal.

The ensemble spectra averaged over all 60 realizations for each of the considered spectral slopes are shown in Fig 2F, all them displaying the desired *β* value to within 0.05 error margin. Extensive descriptions, demonstrations and applications to different geophysical properties of these fractal generation algorithms can be found for example in [19, 41–43].

## Results

### Multiscale analysis of synthetic fractal time-series

The hypothesized relationship between *MSE* and the fractal scaling behavior (Eq 7) implies that the slope of MSE against *τ* in a log-log plot is equal to *H*. Taking into account the definition of complexity provided by [20, 26, 27]—if for the majority of the scales the entropy values are higher for one time-series than for another, the former is considered more complex than the latter—then three different regimes of complexity emerge for Eq (7): (i) when *β* = 1, i.e. 1/f noise with *H* = 0, entropy should be constant with scale; (ii) when *β* < 1 entropy should decrease with increasing *τ*; and (iii) when *β* > 1 entropy should increase with increasing *τ*. This transition at *β* = 1 contains information about the degree of stationarity of the field (e.g. [38, 44]): in time-series with *β* < 1 the process is stationary, while for time-series with *β* > 1 the process is non-stationary but with stationary increments.

**Fig 3:**
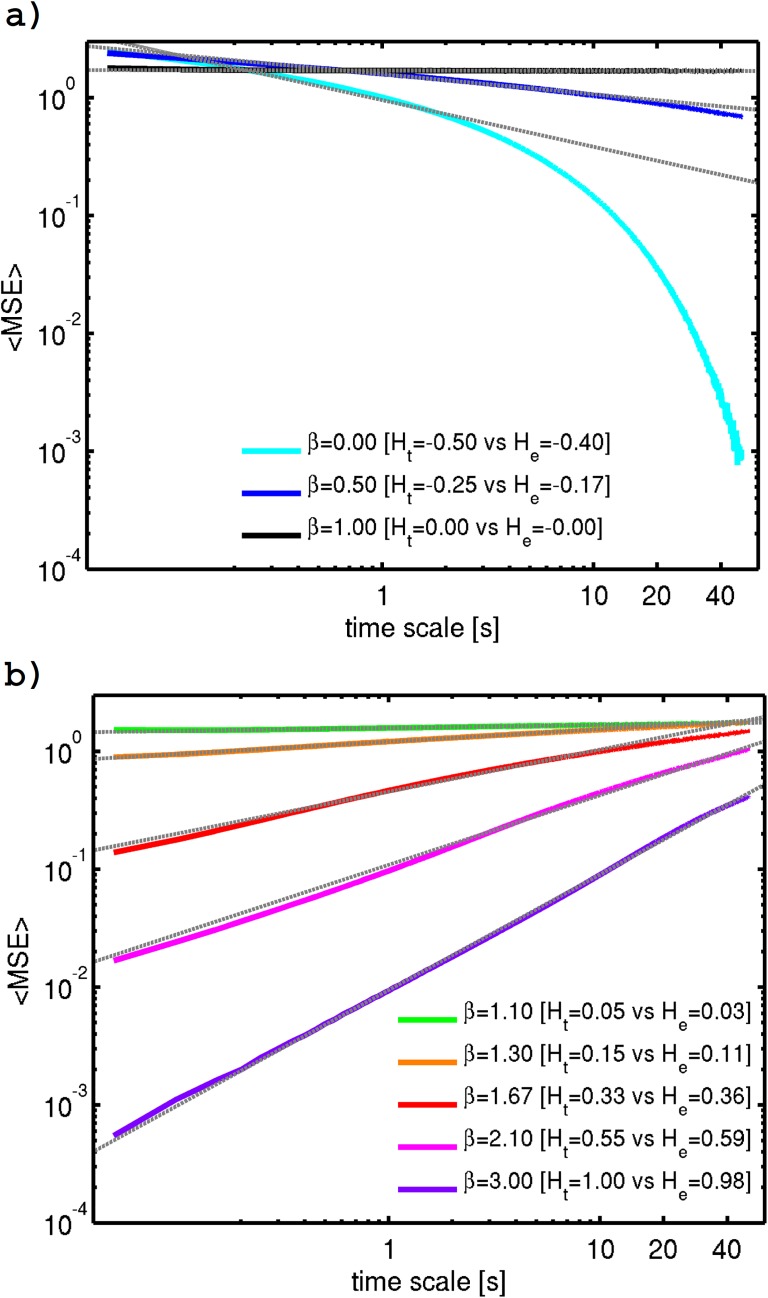
Ensemble MSE averaged over the 60 SF realizations for each of the considered *β* values. a) *β* values of 0, 0.5 and 1.0; and b) *β* values of 1.1, 1.3, 5/3, 2.1, and 3.0. The corresponding *H* values–theoretical (from Eq 4) and empirical (estimated by linear regression, with the fits shown by gray dashed lines) are detailed in the legend. Here the ensemble average is taken over the 60 SF realizations for each of the considered *β*.

*MSE* analysis of the SF time-series (Fig 3) reveals that Eq (7) is verified over a wide range of scales for all spectral exponents typically found in geophysical fields (0 < *β* < 3). The results also confirm the three distinct regimes separated by the *β* = 1 transition. Additionally, in agreement with previous empirical and theoretical results [20, 26, 27], maximum entropy at the smallest scale occurs for random noise (*β* = 0), but maximum complexity occurs at *β* = 1 (1/f noise), since in the former *MSE* decreases rapidly with increasing time-scale (∝ *τ*^−0.5^), while in the latter *MSE* is constant with temporal scale. This result can be explained by the fact that 1/f noise contains complex structures (correlations) across multiple time scales whereas random noise does not.

Notice that the existence of breaks in the power-law (scaling) behavior of *MSE* for large temporal scales in Fig 3 (particularly for lower *β* values, e.g. for *β* = 0) is in disagreement with the Fourier spectral scaling behavior which holds to a remarkable approximation over this entire scale range (Fig 2F). We argue that this break in *MSE* should be a spurious artifact, due to recognized limitations of the *MSE* algorithm such as the rapid decrease in sample number with increasing coarse-graining scale and its dependence in the power of the signal (see e.g. [29, 45]).

### Multiscale analysis of high-frequency wind time-series

**Fig 4:**
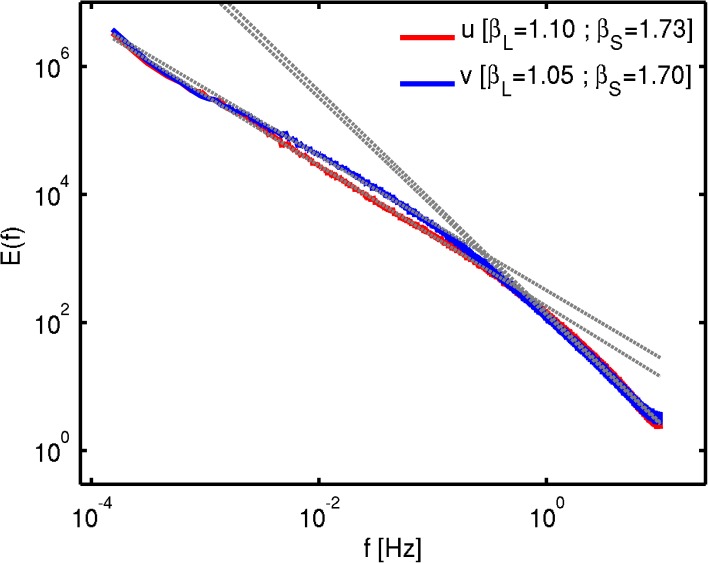
Ensemble Fourier power spectrum computed over all available ABL wind time-series realizations in the CASES-99 valid dataset. The red line represents the zonal wind component (u) and the blue line represents the meridional wind component (v).

Zonal and meridional ensemble wind power spectra were obtained by averaging over all the available time-series realizations in the valid observational CASES-99 dataset (Fig 4). Both horizontal wind components display nearly identical ensemble averaged temporal scaling behavior, with two scaling regimes separated by a scaling break occurring approximately between 1 and 4 seconds. The high-frequency scale range displays a nearly constant 5/3 scaling behavior in agreement with Kolmogorov’s predictions for the inertial range. The deviations from 5/3 scaling are smaller than 0.1, hence smaller than the typical ~0.2 errors in *β* estimations (e.g., [46]). The low-frequency scale range displays spectral exponent values of approximate 1–1.1, close to the often reported *β*∼1 scaling regime (as discussed in Section 1).

**Fig 5:**
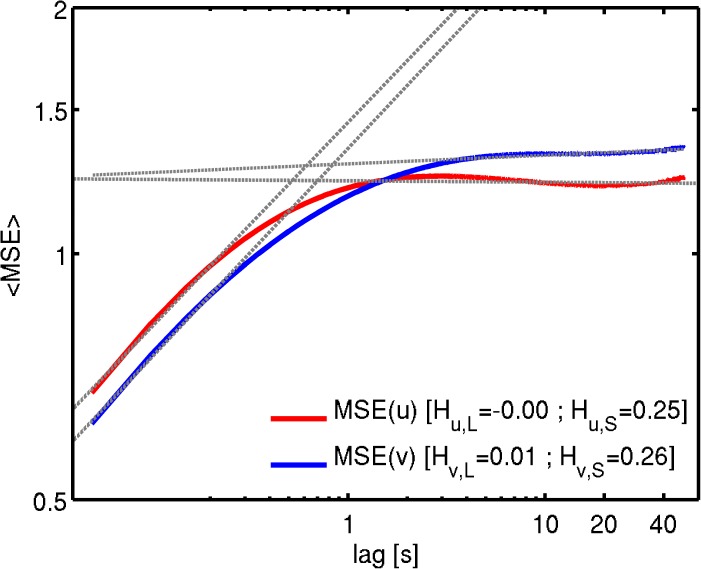
Log-log plots of ensemble averaged *MSE* computed from all the valid CASE-99 time-series for zonal wind (red) and meridional wind (blue). The dashed grey lines are the linear regression fits, with the corresponding slopes identified in the legend.

Log-log plots of *MSE* against *τ* (Fig 5) show a consistent picture with the Fourier analysis for both zonal and meridional wind components: a slope around 0.25–0.26 at the small-scales, close to the expected value *H* = 1/3; then a scaling break occurring around 1s; and a slope ≈ 0 at the large-scales, also in agreement with the spectral slopes estimated from Fig 4. Hence equivalency of the power law exponents between *MSE* and fractal scaling are confirmed by the high-frequency near-surface wind observational data. It is important to point out that the inertial scale range in the CASES-99 dataset spectra is quite narrow (extending only over about one order of magnitude) and may be contaminated by measurement errors. Nonetheless, the scaling behavior found is similar to the expected, and often reported, Kolmogorov 5/3 (within a 0.08 error margin) scaling and the results are consistent between the MSE and spectral analysis, and hence the result are considered to be robust.

This equivalency between *MSE* and scaling behavior is also is consistent with the phenomenological “energy cascade”, where the viscous dissipation works as an information sink at the small-scale end of the inertial range, hence fluctuations are dissipated and information is destroyed at the dissipation scale range. Then inside the inertial range, the average fluctuations (see Eq 2) and the rate of information creation increase with increasing time-scale following a nearly 1/3 power-law up to scales of about 1 second, where a break in the scaling behavior occurs. A new regime with *H* close to zero emerges at the larger scales, or in other words a 1/f behavior is approached in the energy-containing scales. This corresponds to a widespread information creation at the larger scales consistent with the multiple forcings that occur over a vast range of scales in the ABL (e.g. interaction with topography, surface fluxes, etc.). Notice also that the nearly constant value of *MSE* ≈ 1.7 obtained at the larger-scales is in close agreement with previous investigations of [20, 27] where a constant *MSE* = 1.8 was obtained both empirically and theoretically for 1/f signals.

### Multiscale analysis of hourly wind time-series

**Fig 6:**
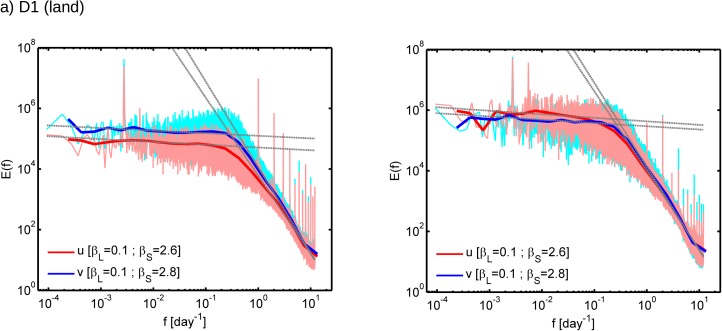
Ensemble Fourier power spectrum of CFSR hourly 10m wind speed computed over (a) D1 (land) and (b) D2 (ocean) sub-domains (see Fig 1). The pink lines represent the zonal wind component (u) and the red lines represent the respective logarithmic averaged spectrum. The light blue lines represent the meridional wind component (v) and the red line represents the respective logarithmic averaged spectrum. The dashed grey lines are the linear regression fits, with the corresponding slopes identified in the legend.

The spectral analysis is repeated for CFSR zonal and meridional near-surface wind hourly time-series. The ensemble spectra are computed for each of the considered sub-domains in Fig 1. The results show that, unlike the sub-hourly spectra from CASES-99 data-set, here two major energy peaks arise at specific time-scales both over land and ocean (Fig 6): the left-most peak corresponds to the yearly cycle and the first peak after the scaling break corresponds to diurnal cycle. The other peaks are the respective harmonics. Over the sea both yearly and daily peaks have smaller amplitude compared to their counterparts over land. This is a consequence of the more intense response of land surface to the radiative forcing, as compared to the ocean where there is a higher thermal inertia.

Before computing the spectral exponents using linear regression, the frequencies most affected by yearly and diurnal peaks were removed and logarithmic averaging was performed to the spectra to avoid fluctuations. The results show similar scaling behavior at temporal scales between peaks for land and ocean (Fig 6): both display an approximately isotropic scaling behavior with *β* between 2.6 and 2.8 at sub-daily time-scales Then there is a break in the scaling between 1 and about 10 days and at the larger scales there is an abrupt flattening of the power spectrum which becomes nearly horizontal with *β* values around 0.1 in both zonal and meridional directions for land and ocean. As discussed in Section 1, this nearly horizontal region of the power spectrum has been reported in several other previous investigations. Our results do not show any significant differences in the low-frequencies slope between ocean and land, with the differences within the 0.2 error margin typically found for spectral exponent estimation [46].

The noisier structure of the CFSR wind power spectra as compared to the high-frequency CASES-99 data suggests that large spatial coherency of the Fourier phase exists inside each of the considered sub-domains. Notice that the re-analysis products are based on numerical models with coarse-grain grid-boxes. Thus important differences are expected when compared to point measurements. In particular the considerable smoothing introduced by the strong spatial averaging introduced by finite grids and numerical diffusion significantly decreases spatial heterogeneity.

**Fig 7:**
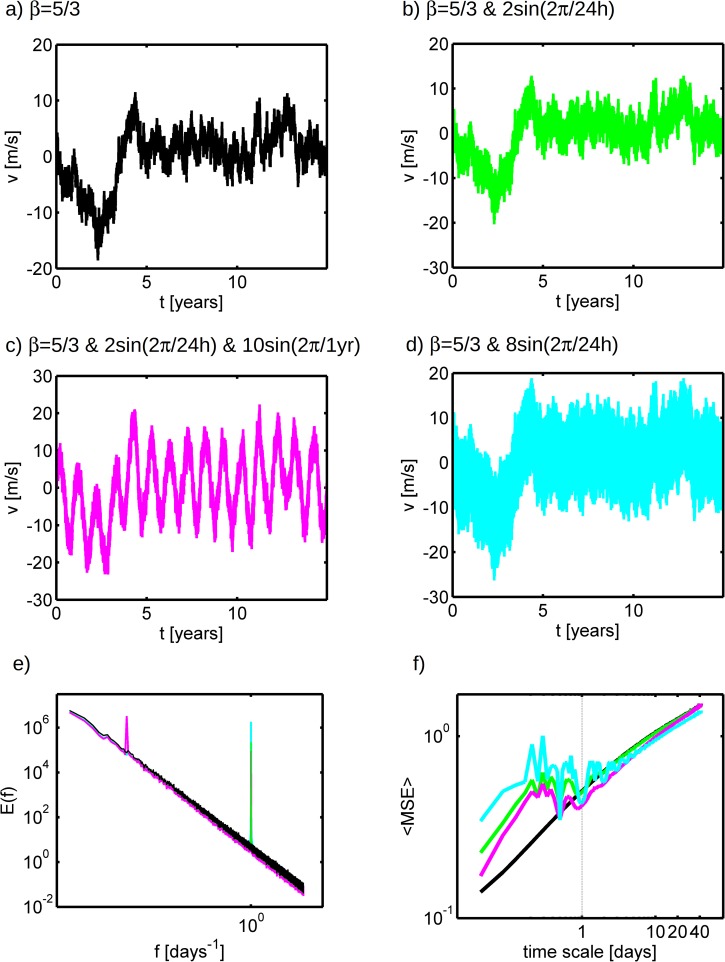
Synthetic fractal wind time-series with a) *k*^−5/3^ spectrum; b) same as a) but added a 2 sin(2*π*/24*h*) term; c) same as a) but added a 2 sin(2*π*/24*h*) and a 10 sin(2*π*/1*yr*) terms; d) the respective power spectra for the time-series in a) (black), b) (green) and c) (pink); e) the respective log-log *MSE* against time-scale plot for the time-series in a) (black), b) (green) and c) (pink). The grey dashed line represents the daily scale.

The existence of characteristic time-scales associated with the large daily and yearly spectral peaks does not invalidate the underlying scaling behavior. In fact, this type of peaks are expected for measurements close to the surface in the atmospheric boundary layer due to surface interactions that can cause accumulation of energy at specific scales, as discussed in [2]. The importance of the underlying scaling behavior is illustrated by generating three new sets of SF time-series, all of them with *β* = 5/3 but with a superposed high amplitude sinusoidal signals: the first has a daily peak (Fig 7B), the second has both daily and yearly peaks (Fig 7C), the third has a higher amplitude daily peak (Fig 7D). These time-series are compared against a pure 5/3 SF time-series (Fig 7A). The results clearly show that power spectra are not significantly modified in any of the cases by the addition of the periodic signals, except at the particular time-scales forced by the periodic terms (Fig 7E). But the time-series themselves (Fig 7B–7D) are quite different from a simple periodic signal since they display significant variability over a wide range of time-scales, particularly large variability of the mean is found for fractal time-series.

Unlike the Fourier analysis, the *MSE* algorithm applied here (and commonly applied in many studies) based on coarse-graining of a time-series is clearly affected by the introduction of periodic signal outside the scales of forcing (Fig 7F). While only slight reductions of entropy are obtained at the forcing scales and its harmonics (expected due to increased periodicity of the signal), there is a clear entropy accumulation at the small scales which is not expected. Hence, in the present formulation, *MSE* should be used with care to quantify the scaling exponents of time-series with strong superposed periodic components. These results suggest that removal of the periodic peaks might be required, but this hypothesis is left for future work since the main objective the main goal here is to explore the links between the current *MSE* formulation and fractal behavior.

**Fig 8:**
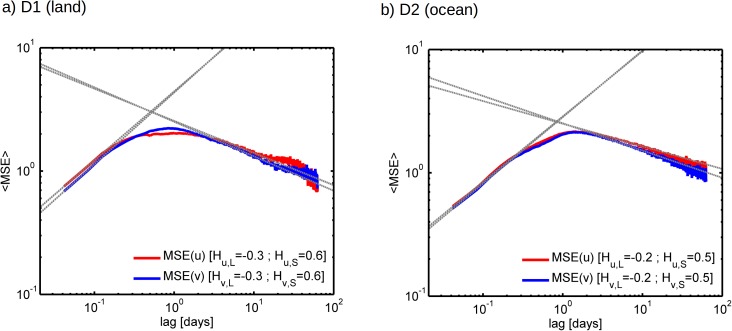
Log-log plots of ensemble averaged *MSE* computed from CFSR hourly time-series over sub-domain a) D1 and b) D2. The red lines correspond to zonal wind and the blue lines correspond to meridional wind. The dashed grey lines are the linear regression fits, with the corresponding slopes identified in the legend.

Finally, the *MSE* analysis of the CFSR wind time-series is presented in Fig 8. As expected from the results presented in Fig 7, the *MSE* analysis of time-series with strong periodic components is less accurate in predicting the underlying fractal exponents due to the presence of strong superposed periodic signals. Also in agreement with Fig 7, at the large scales the errors are lower than at the small scales. Over land the error in *H* at the large-scales is around 0.15 for both components while at the small-scales the error is around 0.2 for zonal wind and 0.3 for meridional wind. Over the ocean, at the large-scales the error is around 0.15 for zonal wind and around 0.25 for meridional wind, while at the small-scales it is 0.35 for the zonal wind and 0.4 for the meridional wind. Despite these increased errors in the slopes, the main feature of the power spectra that occurs both over land and ocean is reproduced by the MSE analysis: the existence of a pronounced scaling break at scales of a few days separating a high-frequency regime with positive *H* values from a low-frequency regime with negative *H* values. This opens a new interpretation of the wind power spectral shape. At the sub-daily scales there is an increase of the information being created (entropy) with scale. Then there is a local maximum of information being created at scales between about 1 and 10 days which is associated with the occurrence synoptic weather features. At larger-scales, from about 10 days to nearly 1 decade, there is a decrease of the amount of created information with scale. On top of this, two periodic signals are superposed at daily and yearly time-scales.

It is likely that the high *β* values found for the sub-daily scales on CFSR data are being overestimated. This is due to the fact that CFSR re-analysis is based on a numerical model with a coarse finite grid resolution. Generally this type of models is overly diffusive at the small-scales due to excessive numerical filtering. This has been previously shown for different models, resolutions and variables by [19, 47].

## Summary and conclusions

Here, the link between fractal scaling behavior of a time-series and its *MSE* variation with time-scale, previously suggested by [36, 37], is explored for the first time in atmospheric fields providing a new independent empirical validation of this relationship. Additionally, the promising path opened by the *MSE*-fractal power-law behavior relationship for interpretation of the scaling behavior based on the information theory is explored for the first time in the atmosphere, providing insight onto the scaling behavior of ABL wind and guiding more fruitful future applications of *MSE* in diverse fields where complex signals with fractal scaling behavior emerge. This type of exact correspondences between seemingly different concepts play important role in all fields of physics.

*MSE* displays power-law dependence on the temporal scale (*MSE* ∝ *τ*^*H*^) where the exponent *H* is equal to the fractal scaling exponent obtained from the first-order structure function, which is also directly related to the fractal spectral exponent by *β* = 1 + 2*H*. In the present work this direct link between *MSE* and scaling behavior was successfully verified for a set of synthetic time-series covering the entire range of spectral exponents found for real atmospheric signals 0 ≤ *β* ≤ 3. The *MSE*-fractal relationship was also verified for both horizontal components of near-surface wind observations obtained from the CASES-99 experiment. The analysis of this high-frequency (20Hz) time-series reveals the existence of an inertial range at scales below about 1 s with *β* ≈ 5/3 in agreement with Kolmogorov’s 1941 [4] predictions. At the larger-scales there is scaling transition and a *β* ≈ 1.1 regime emerges. This spectral shape is found to be nearly identical for both zonal and meridional wind components. *MSE* reveals a remarkably similar behavior, thus verifying the proposed *MSE*-fractal relationship.

As pointed out in Section 1, several previous works have reported a similar transition at scales on the order of 1 s, while others find no scaling break up to a few days. The 5/3 scaling inside the inertial range of wind fields seems to be a universal property. However the intermediate scales outside the inertial range do not possess the same degree of universality. There is no consensus on the large-scale end of the inertial range nor on the value of the scaling exponent at these larger-scales. This variability may be caused by the sensitivity of near-surface variables to the underlying surface forcing and also to the particular atmospheric conditions, which can modify the energy distribution across scales [17–19, 43, 48, 49].

The hourly near-surface wind time-series from CFSR re-analysis reveals that at even larger temporal scales there are two localized high amplitude spectral peaks at daily and yearly scales. While the superposition of these periodic signals has an important role in modulating the time-series, our results show that it doesn’t destroy the underlying scaling behavior which still plays an important role in the observed signal. The CFSR wind power spectra also reveals another scaling break, occurring at scales between about 1 and 10 days over both land and ocean and in both horizontal wind components. This transition corresponds to an abrupt flattening of the power spectrum towards the large-scales, in clear contrast with the steeper slopes observed at the sub-daily scales.

The MSE-fractal relationship implies that three distinct cases exist. When *β* = 1, *MSE* is constant with scale, i.e. the average rate of creation of new information and the magnitude of the average fluctuation is the same at all temporal scales. This is the case with maximum (multi-scale) complexity. The complexity decreases as the spectral exponent deviates from 1, but with different behaviors for *β* > 1 and *β* < 1. When *β* < 1, the average fluctuation decreases with increasing scale which is equivalent to a decrease in the average rate of creation of new information (and uncertainty). When *β* > 1, the average fluctuation increases with increasing scale which is equivalent to an increase in the average rate of creation of new information (and uncertainty) with increasing scale.

The application of this MSE-fractal relationship to the near-surface wind provides a path for interpretation of its spectra based on the information theory. At the sub-hourly time-scales, this interpretation is consistent with the phenomenological `energy cascade', where the inertial range transfers turbulent energy from the large (energy-containing) scales to the dissipation range. The smoothing introduced by the viscous dissipation at very small scales (~ 1 ms) is expected to cause a minimum of the average fluctuations (measured by the first order structure function) and equivalently on the rate of information creation (measured by entropy). In other words, the viscous dissipation is an information sink. At the low-frequency end of the inertial range, the 5/3 scaling is broken and a new temporal scaling regime emerges, with a tendency towards a 1/f (*β* ≈ 1). This regime corresponds to a widespread information creation, which is consistent with the multiple forcings that occur over a vast range of scales in the ABL (e.g. interaction with topography and surface fluxes).

The analysis of hourly wind time-series reveals yet another break in the scaling behavior, corresponding to a local maximum of information being created at scales between about 1 and 10 days. These time-scales are associated with the occurrence synoptic features, known to dominate weather patterns. At even larger-scales, from about 10 days to nearly 1 decade, there is a sign inversion and the amount of new information created with increasing scale is now decreasing. This corresponds to the the flatter low-frequency portion of the spectrum, which has been reported by numerous previous investigations based on different near-surface wind datasets. This suggests that, just like the existence of a 5/3 inertial range, the low-frequency weather should be a universal property of near-surface wind. In contrast, the widespread of scaling exponents and scaling intervals found in the intermediate scales (from a seconds to a few days) suggest that the mesoscale variability of ABL winds should be strongly dominated by the particular conditions and bottom boundary forcings.

As a final note, our results point out a decrease in information creation at scales between about 10 days and a decade. Theoretically, in the light of Shannon’s information entropy, this corresponds to a decrease in the uncertainties at these scales. However, this does not translate into actual increased accuracy of the medium and long-range predictions obtained from numerical models because the atmosphere is a chaotic system, where exponential growth of errors can occur and, consequently, the predicted solution will quickly diverge from reality. This is a well known fact ever since Lorenz’s 1963 seminal paper [50].

## References

[1] Tuck AF. From molecules to meteorology via turbulent scale invariance. Quart. J. Roy. Meteor. Soc. 2010; 136: 1125–1144.

[2] Lovejoy S, Schertzer D. The Weather and Climate: Emergent Laws and Multifractal Cascades. Cambridge University Press; 2013.

[3] Veneziano D, Langousis A, Furcolo P. Multifractality and rainfall extremes: A review. Water Resour. Res. 2006; 42, w06D15.

[4] Kolmogorov A. Local structure of turbulence in an incompressible liquid for very large Reynolds numbers. Proc. Acad. Sci. USSR Geochem. 1941; 30: 299–303.

[5] Tchiguirinskaia I, Schertzer D, Lovejoy S, Veysseire J. Wind Extremes and Scales: Multifractal Insights and Empirical Evidence In: Peinke J, Schaumann P, Barth S, editors. Wind Energy. Springer Berlin Heidelberg; 2007 pp. 99–104.

[6] Muzy JF, Baile R, Poggi P. Intermittency of surface-layer wind velocity series in the mesoscale range. Phys. Rev. E. 2010; 81–056308. 10.1103/PhysRevE.81.056308 20866323

[7] Lovejoy S, Schertzer D. Low-Frequency Weather and the Emergence of the Climate In: Sharma AS, Bunde A, Dimri VP, Baker DN, editors. Extreme Events and Natural Hazards: The Complexity Perspective. American Geophysical Union, Washington, D. C. 2012.

[8] Schmitt F, Schertzer D, Lovejoy S, Brunet Y. Estimation of universal multifractal indices for atmospheric turbulent velocity fields. Fractals. 1993; 01: 568–575.

[9] Schmitt F, Schertzer D, Lovejoy S, Brunet Y. Empirical study of multifractal phase transitions in atmospheric turbulence. Nonlinear Process. in Geophys. 1994; 1: 95–104.

[10] Schmitt F. Gusts in intermittent wind turbulence and the dynamics of their return times In: Peinke J, Schaumann P, Barth S, editors. Wind Energy. Proceedings of the Euromech Colloquium. Springe 2007; pp. 73–79.

[11] Calif R, Schmitt F. Modeling of atmospheric wind speed sequence using a lognormal continuous stochastic equation. J. Wind Eng. Ind. Aerodyn. 2012; 109: 1–8.

[12] Calif R, Schmitt F. Multiscaling and joint multiscaling description of the atmosphericwind speed and the aggregate power output from a wind farm. Nonlinear Process. in Geophys. 2014; 21: 379–392.

[13] Fu Z, Li Q, Yuan N, Yao Z. Multi-scale entropy analysis of vertical wind variation series in atmospheric boundary-layer. Commun. Nonlinear Sci. Numer. Simulat. 2014; 19: 83–91.

[14] Lovejoy S, Schertzer D. Towards a new synthesis for atmospheric dynamics: Space–time cascades. Atmos. Res. 2010; 96(1): 1–52.

[15] Huybers P, Curry W. Links between annual, Milankovitch and continuum temperature variability. Nature. 2006; 441: 329–332. 10.1038/nature04745 16710417

[16] Katul G, Chu CR, Parlange MB, Albertson JD, Ortenburger TA. Low-wavenumber spectral characteristics of velocity and temperature in the atmospheric surface layer. J. Geophys. Res.: Atmos. 1995; 100: 14243–14255.

[17] Katul G, Chu CR. A theoretical and experimental investigation of energy-containing scales in the dynamic sublayer of boundary-layer flows. Boun.-Layer Meteor. 1998; 86: 279–312.

[18] Lauren M, Menabde M, Seed A, and Austin G. Characterisation and simulation of the multiscaling properties of the energy-containing scales of horizontal surface-layer winds. Bound.-Layer Meteor. 1999; 90, 21–46.

[19] Nogueira M, Barros AP. The nonconvective/convective structural transition in stochastic scaling of atmospheric fields. J. of Geophys. Res.: Atmos. 2014; 119: 13771–13794.

[20] Costa M, Goldberger AL, Peng CK. Multiscale Entropy Analysis of Complex Physiologic Time Series. Phys. Rev. Lett. 2002; 89: 068102 10.1103/PhysRevLett.89.068102 12190613

[21] Shannon CE. Mathematical Theory of Communication. Bell Syst. Tech. J. 1948; 27: 379–423.

[22] Jaynes ET. Information Theory and Statistical Mechanics. Phys. Rev. 1957; 106: 620–630.

[23] Sinai YG. On the notion of entropy of a dynamical system. Dokl. Akad. Nauk. 1959; 124: 768–771.

[24] Pincus SM. Approximate entropy as a measure of system complexity. Proc. Natl. Acad. Sci. 1991; 88: 2297–2301. 11607165 10.1073/pnas.88.6.2297 PMC51218

[25] Richman JS, Moorman JR. Physiological time-series analysis using approximate entropy and sample entropy. Am. J. Physiol. Heart. Circ. Physiol. 2000; 278: H2039–H2049. 10843903 10.1152/ajpheart.2000.278.6.H2039

[26] Zhang YC. Complexity and 1/f noise. A phase space approach. J. Phys. I. 1991; 1(7): 971–977.

[27] Costa M, Goldberger AL, Peng CK. Multiscale entropy analysis of biological signals. Phys. Rev. E. 2005; 71: 021906. 10.1103/PhysRevE.71.021906 15783351

[28] Crutchfield JP. Between order and chaos. Nature Phys. 2012; 8: 17–24.

[29] Humeau-Heurtier A. The Multiscale Entropy Algorithm and Its Variants: A Review. Entropy. 2015; 17: 3110–3123.

[30] Ahmed MU, Mandic DP. Multivariate multiscale entropy: A tool for complexity analysis of multichannel data. Phys. Rev. E. 2011; 84(6): 061918–1–061918–10. 10.1103/PhysRevE.84.061918 22304127

[31] Li H, Meng Q, Wang Y, Zeng M. Multi-scale Entropy Analysis of Single-point Wind Speed in Outdoor near-surface Environments. IEEE Int. Conf. Elec. Control Eng. 2011, pp. 4579–4582

[32] Balzter H, Tate NJ, Kaduk J, Harper D, Page S, Morrison R, Muskulus M, Jones P. Multi-Scale Entropy Analysis as a Method for Time-Series Analysis of Climate Data. Climate. 2015; 3: 227–240.

[33] Li Z, Zhang YK. Multi-scale entropy analysis of Mississippi River flow. Stoch. Environ. Res. Risk Assess. 2008; 22:

[34] Chou CM. Applying Multiscale Entropy to the Complexity Analysis of Rainfall-Runoff Relationships. Entropy. 2012: 14: 945–957.

[35] Zhou Y, Zhang Q, Li K, Chen X. Hydrological effects of water reservoirs on hydrological processes in the East River (China) basin: complexity evaluations based on the multi-scale entropy analysis. Hydrol. Process. 2012; 26: 3253–3262.

[36] Courtiol J, Perdikis D, Petkoski S, Müller V, Huys R, Sleimen-Malkoun R, et al The multiscale entropy: Guidelines for use and interpretation in brain signal analysis. J. Neuro. Meth. 2016; 273: 175–190. 10.1016/j.jneumeth.2016.09.004 27639660

[37] Gao J, Hu J, Liu F, Cao Y. Multiscale entropy analysis of biological signals: a fundamental bi-scaling law. Front. Comput. Neurosci. 2015; 9: 64 10.3389/fncom.2015.00064 26082711 PMC4451367

[38] Schertzer D, Lovejoy S. Physical Modeling and Analysis of Rain and Clouds by Anisotropic Scaling Multiplicative Processes. J. Geophys. Res.: Atmos. 1987; 92: 9693–9714.

[39] Poulos GS, Blumen W, Fritts DC, Lundquist JK, Sun J, Burns SP, et al CASES-99: A Comprehensive Investigation of the Stable Nocturnal Boundary Layer. Bull. Amer. Meteor. Soc. 2002; 83: 555–581.

[40] Saha S, Moorthi S, Pan H, Wu X, Wang J, Nadiga S, et al The NCEP Climate Forecast System Reanalysis. Bull. Amer. Meteor. Soc. 2010; 91: 1015–1057,

[41] Bindlish R, Barros AP. Aggregation of digital terrain data using a modified fractal interpolation scheme. Comput. Geosci. (UK). 1996; 22, 907–917.

[42] Bindlish R, Barros AP. Disaggregation of rainfall for one-way coupling of atmospheric and hydrological models in regions of complex terrain, Global Planet. Change. 2000; 25(12): 111–132.

[43] Nogueira M, Barros AP. Transient stochastic downscaling of quantitative precipitation estimates for hydrological applications. J. Hydrol. 2015; 529(3): 1407–1421.

[44] Kirkby MJ. The fractal geometry of nature Benoit B. Mandelbrot. W. H. Freeman and co., San Francisco 1982; pp. 460.

[45] Valencia JF, Vallverdú M, Schroeder R, Voss A, Vázquez R, Bayés de Luna A, et al Complexity of the Short-Term Heart-Rate Variability Using Entropy Rates to Improve Risk Stratification to Predict Cardiac Mortality. IEEE Eng. Med. Bio. Mag. 2009; 28: 72–78. 19914891 10.1109/MEMB.2009.934621

[46] Barros AP, Kim G, Williams E, Nesbitt SW. Probing orographic controls in the Himalayas during the monsoon using satellite imagery. Nat. Hazards Earth Syst. Sci. 2004; 4: 29–51.

[47] Skamarock W. Evaluating mesoscale NWP models using kinetic energy spectra. Mon. Weather Rev. 2004; 132: 3019–3032.

[48] Katul G, Porporato A, Nikora V. Existence of k −1 power-law scaling in the equilibrium regions of wall-bounded turbulence explained by Heisenberg’s eddy viscosity. Phys. Rev. E. 86: 066311. 10.1103/PhysRevE.86.066311 23368042

[49] Nogueira M, Barros AP, Miranda PM. Multifractal properties of embedded convective structures in Atmospheric orographic precipitation: Atmospheric toward subgrid-scale predictability. Nonlinear Process. Geophys. 2013; 20, 605–620.

[50] Lorenz E. Deterministic Nonperiodic Flow. J. Atmos. Sci. 1963; 20: 130–141.

